
JOURNAL INFORMATION
==============================
NLM Title Abbreviation: Atlanta Med Surg J (1884)
NLM Journal ID: 101251623

Atlanta Medical and Surgical Journal (1884)

Publisher: Biomedical Journal Digitization Project

ARTICLE INFORMATION
==============================
PMCID: PMC9199297
PMID: 35829594
Article version: 1
Subjects: Original Communications

The Possibilities of Perfection in Aseptic Surgical Technique* Read by title before the Georgia Medical Association.

Davis E. C. A.B., M.D. Atlanta, Ga.; Lecturer on Diseases of Children, Southern Medical College, Atlanta

Print publication date: 1898 Dec
Volume: 15
Volume Series: New Ser
Issue: 10
First page: 649
Last page: 657
License: This work is free of known copyright restrictions. For more information, please see PMC Back Issue Digitization.
License URL: https://creativecommons.org/publicdomain/mark/1.0/

==============================

ATLANTA

Medical and Surgical Journal.

Vol. XV. DECEMBER, 1898. No. 10.

DUNBAR ROY, A.B., M.D., EDITOR.

M. B. HUTCHINS, M.D., BUSINESS MANAGER.

### ORIGINAL COMMUNICATIONS.

### THE POSSIBILITIES OF PERFECTION IN ASEPTIC SURGICAL TECHNIQUE.*

By E. C. DAVIS, A.B., M.D.,

Lecturer on Diseases of Children, Southern Medical College, Atlanta.

In appearing before this Association to present a paper upon such a subject as has been chosen I almost feel inclined to introduce it with an apology, for I cannot promise anything new, and fear the substance of this paper may seem trite and commonplace to many. I am encouraged, however, in my efforts by the knowledge that while it may prove uninteresting to some, to others, now familiar with the contents, it may be a review of the details in such a manner as to at least interest them. I will also mention that many of my opportunities for investigating this subject have been due to the counsel of Dr. J. B. S. Holmes and the advan-

*Read by title before the Georgia Medical Association.

tages accruing from assisting him in his surgical operations at his private hospital for the past three and one half years.

The developmental stages through which aseptic surgery has passed are extremely interesting, but would consume too much time to enter upon their consideration extensively; it would not be out of place, however, to mention that John Hunter, when he called attention to the differences in behavior of subcutaneous injuries and those in which the skin was broken, was verging close upon the principles which control the great field of operative surgery to-day; and when Malgaigne, profiting by this observation, performed his operations upon the tendons subcutaneously without having suppuration, more thought was still directed to this subject. It remained, however, for Lister to put into practical use the suggestions formulated by Pasteur and other eminent workers in fermentative changes. He, however, believed infection to be conveyed largely through the atmosphere, and to prevent this kept the carbolic spray in active use during operations. His ideas were at first greatly ridiculed by the profession at large, but his results proved conclusively the improvement which he had made, and when Robert Koch demonstrated the micro-organisms producing infection, and made cultures of them, the link of evidence was complete, and the attention of investigators was turned to other media of conveying these micro-organisms than the air. It was found that the air contained but a small number of germs compared with a mass of decomposing matter, and a drop of pus was found fairly teeming with them, containing in fact several millions. Again many of the germs found in the air were innocuous, while those transported by contact were more often infectious. The discussion concerning the cause of infection is but an interesting incident in history, as to-day all accept the bacterial origin of infection, although some of the English surgeons were loath to yield to this belief, preferring to call this dirt rather than bacteria. When it was found that contact was the most frequent source of infection, and that carbolic acid was a very weak destructive agent to these germs, then Koch constructed the following, showing the effect of different chemical agents upon the growth of micro-organisms:

| | Growth was distinctly hindered by the proportion of: | | Growth was completely put a stop to by the proportion of: |
|---|---|---|---|
| Bichloride of mercury | 1:1,600,000 | | 1:300,000 |
| Oil of mustard | 1: 333,000 | | 1: 33,000 |
| Arsenate of potassium | 1: 100,000 | | 1: 10,000 |
| Thymol | 1: 80,000 | | |
| Oil of turpentine | 1: 75,000 | | |
| Osmic acid | 1: 6,000 | | |
| Oil of cloves | 1: 5,000 | | |
| Potash soap | 1: 5,000 | | 1: 1,000 |
| Iodine | 1: 5,000 | | |
| Salicylic acid | 1: 3.300 | | 1: 1,500 |
| Hydrochloric acid | 1: 2,500 | | 1: 1,700 |
| Camphor | 1: 2.500 | over | 1: 1,250 |
| Eucalyptol | 1: 2,500 | over | 1: 1,000 |
| Borax | 1: 2,000 | | 1: 700 |
| Benzoic acid | 1: 2,000 | | |
| Bromine | 1: 1,500 | | |
| Chlorine | 1: 1,500 | | |
| Permanganate of potassium | 1: 1,400 | | |
| Boric acid | 1: 1,250 | | 1: 800 |
| Carbolic acid | 1: 1,250 | | 1: 850 |
| Quinine | 1: 830 | | 1: 625 |
| Chlorate of potassium | 1: 250 | | |
| Benzoate of sodium | 1: 200 | | |
| Alcohol | 1: 100 | | 1: 125 |
| Chloride of sodium | 1: 64 | | |

Within recent years there has not been given a sufficient amount of attention to the condition of the patient as a causative factor in infection. But there are no surgeons doing active work without observing that the conditions of certain patients seem to conduce to suppurative changes, while others resist the invasions to a surprising degree. This is especially noticeable in some of the diseased conditions, viz., diabetes and such diseased conditions as lead to a marked retrograde metamorphosis of the tissues. The patient's surroundings may also contribute its quota to infection, but the causes which are the most operative, and the ones which to-day stand paramount, are the conveyance of infection through lack of cleanliness of the patient, and especially that portion of the patient where the incision is to be made and its immediate neighborhood; personal cleanliness of the surgeon and his assistants; clean instruments and dressing; and more especially clean hands. From a theoretical standpoint the attainment of these essentials is readily possible, but when we apply them practically we encounter difficulties which occasionally lead to breaks in our asepsis in spite of all our care and attention. To avoid infection we must keep clearly in mind the conditions necessary for its production, that we may at least be enabled to withdraw one or more of these conditions in handling wounds. The conditions necessary for infection are that we must have: 1. The presence of the micro-organisms capable of producing infection; 2. The proper pabulum for its growth and development; 3. Suitable temperature for its growth and reproduction; 4. A certain amount of moisture. Based upon these conditions are all the rules of aseptic and antiseptic surgery, and the possibilities of withdrawing absolutely the first and usually the second conditions are the essentials in the attainment of perfection in aseptic surgical technique. The means of accomplishing these results may, under certain conditions, be extremely simple, while under others are often very complex and difficult. Simplicity in technique should be the aim of all surgeons, but at the same time thoroughness should never be sacrificed in our efforts to appear in favor of too great simplicity. To get rid of the micro-organisms we have two methods of sterilization, viz., chemical and mechanical. By the former we mean the destruction of the micro-organisms by chemical agents, including the various antiseptics which flood the market, and often lure us into a false confidence which may lead to very annoying results. It is extremely difficult to find an agent with sufficient bactericidal influence which will not destroy the tissues when directly applied, or should it not directly destroy the tissues it may seriously impair their functions, so that the invasion of the micro-organisms soon finds an unresisting field and an excellent nidus for its growth and propagation.

It would consume too much time and space to enumerate the various chemicals which are in use and which have been used; the table previously given will show from an experimental standpoint the values of these agents, both as to their ability to destroy and to inhibit micro-organisms. We must not forget, however, that many of these are spore-producing germs, and the spores are much more resistant to destruction than the germs themselves; that which would destroy the germ may have little effect upon its spore, and the spore, under proper surroundings and conditions, develops into an active germ. Chemical sterilization is applied only to those agents or parts to which mechanical is inapplicable, or we may, in a great measure, combine the two when practicable. For instance, in cleansing the operative field, the hands of the surgeon and assistants, these we cannot sterilize by boiling or exposure to sufficiently high temperature to destroy the micro-organisms, hence we must depend upon cleansing by friction and chemical agents. Before calling your attention to the attainment of cleanliness of those parts, I wish first to mention some essentials connected with the preparation of the patient before an operation is begun. In the first place, the patient should be carefully examined as to respiratory, circulatory and excretory organs, also as to habits, in addition to the organs or parts that are to be operated upon. We do this in order that we may determine in the first place whether the patient is a suitable one for an operation, or how well prolonged anesthesia is likely to be borne, and to formulate an idea as to the promptness and completeness of restoration after an operation. The urine should be carefully examined, as should the lungs, heart and blood-vessels, as they not only influence largely the prognosis, but upon this we base our preferences for certain anesthetics. There is no need to prohibit anesthesia in a patient with a distinct cardiac murmur, provided the heart acts regularly, and the volume of the pulse is good, and the blood-vessels' walls are in good condition. We should, however, acquaint the husband or relatives with this condition before administering the anesthetic, in order that no censure may be heaped upon us should a disastrous result follow.

The patient on admission should be given a good bath and clothing changed and then put to bed. A purgative consisting of either castor-oil or salts given and the bowels well emptied. If the case be one requiring an abdominal section, on the night preceding the operation the abdomen should be shaved, and thoroughly cleansed with soap, hot water and brisk scrubbing with nail-brush, followed by a thorough cleansing with alcohol, then covered with a sterile towel, which should be carefully fastened to prevent slipping during sleep. On the morning of the operation the nurse will administer the bath to the patient in bed, following with an alcohol bath.

The patient is anesthetized in the room which she is expected to occupy after the operation, and carried to the operating-room, thereby avoiding the shock produced by watching preparations for the operation, which to a nervous person may amount to a very serious condition. The anesthetist should be one skilled in such administrations, and one in whom the operator imposes great confidence, else his thoughts will be constantly distracted and the operation interrupted and prolonged.

When anesthetized the field of operation and contiguous parts should again be cleansed thoroughly as on preceding night, with the addition of a solution of bichlor. mercury (1–2000th) followed by ether. Before bringing the patient into the operating-room it is advisable to cover the head with a sterile towel; then after the cleansing above described the entire body of the patient, except small space to be operated upon, should be covered with sterile sheet or towels and sterile gauze placed around the field of operation. The sterilization of the instruments and dressings can be accomplished absolutely by the use of heat, either in the form of superheated steam or boiling water, or by dry heat; this latter not yielding as satisfactory results as the other two, owing to its injurious effect upon the cutting instruments, and the lack of penetration into the thicker dressings, etc.

Superheated steam is the plan adopted with most satisfactory results, and the one in use at the Sanatorium of Dr. Holmes. The instruments are boiled in a solution of sodium carbonate one per cent., or better in one half per cent. solution of sodium hydrate, as this will tarnish instruments less than any of the carbonates. They should be boiled in this solution for ten minutes, or should we suspect a contamination from the spore-producing germs, it would be advisable to boil them twice, allowing twenty-four hours to intervene between the boilings, unless we have the superheated steam apparatus which will destroy absolutely the spores as well as the micro-organisms, when exposed for a sufficient length of time.

The instruments should then be transferred to the sterilized vessels in which they are to be kept during the operation; it is best to immerse them with boiled water, or if the surgeon prefers, they may remain in the sterilizer until ready for use, when they may be spread upon sterile towels. Much here depends upon the taste of the operator, whether he likes dry or moist instruments. The dressing should not be exposed to the air until ready for use, as the danger from contamination is greatly increased by exposure.

For ligatures the most satisfactory results have followed the use of silk, for in this we can be sure of perfect sterilization and there is not likelihood of too early absorption. Catgut that is used is sterilized by the cumol method, popularized by Dr. Howard A. Kelly and Dr. Clark of Johns Hopkins, and is undoubtedly sterile, but somehow I early obtained a prejudice against this material, and use it without the same confidence which is given silk. For sutures silkworm gut or silk, sometimes catgut (and this is especially applicable where buried sutures are indicated), in the order named. The silk and silkworm gut can be as safely boiled as the dressing, and can confidently be relied upon when so treated.

We then come to the sterilization of the hands, and here it is that most of the defects in technique occur; there is yet no absolutely certain means of cleansing the hands, and until this is discovered we will always be exposed to risk of infection. The most satisfactory plan so far suggested is that of Dr. Weir, of New York, who recommends first the cleansing of the hands and arms to elbows with soap, water and nail-brush, cleansing the spaces under and around the nails thoroughly, then repeat the cleansing with above-named agents, after which cover the hands and arms with a paste of chlorinated lime and water, to which add several crystals of carbonate of sodium, and again wash. This rapidly liberates the chlorine in a nascent state, which makes it quite active as a germ destroyer, and at the same time leaves a soft, smooth hand. Bichloride of mercury (1–1000th) will usually neutralize or destroy the micro-organisms, but so often leaves a rough, fissured hand that it is being largely discarded by surgeons doing a great deal of operating. Dr. Jos. Price retains quite a fondness for mustard with plenty of soap and friction with good nail-brush. This too leaves a good surgical hand without impairing the tactile sensibilities. I believe after all there is more to be derived from the use of soap, boiled water and a good, stiff brush, used for at least twenty minutes, after the nail spaces around are well cleaned, than from all the chemical agents when applied without previously preparing the hands for their use.

Everything which has not been submitted to the sterilizing process must be considered capable of producing infection, and when touched by the hand should lead to prompt cleansing of the hands again. It is better for the operator and assistants to wear a washable suit or uniform which has just been washed. When any doubt remains about the hands, or when suppurating wounds have been recently handled, it may be advantageous to use rubber gloves that have been sterilized, or cotton gloves that have been boiled.

Some disadvantages which surround the attainment of perfection lie in the difficulties in the preservation of asepsis, even after its primary attainment. We must forcibly impress upon each one present that it is safer to regard every object not sterile as capable of infection, and should it be handled or touched complete sterilization of hands must again be gone through with. Again, operators and assistants are forced often to handle suppurating wounds and to treat infectious diseases, and to make examinations of parts in which bacteria are prevalent; in such cases extra care should be given to the hands and clothing. When infectious diseases are treated it would be advisable to expose the clothing worn to the fumes of formaldehyd, which is used in disinfecting the rooms, then neutralized by exposure to stronger ammonia.

The vessels for sponges, instruments and the irrigators should all be exposed to a sufficiently high temperature for a long enough time to destroy the micro-organisms. If this be impossible, then they should be washed with boiling water, turpentine, bichlor. of mercury (1–1000th), and again with boiling water. In all operative cases we should have just as few assistants as are necessary for the proper performance of the operation, and each assistant should clearly understand what is expected of him and attend strictly to that; we must see that the precautions are thoroughly carried out, superintend the hand cleansing, and, if any doubt exists, take scrapings from hand and nails and inoculate culture tubes, then examine the result; if any show micro-organisms of suppuration, then debar this assistant from the operating-room for a specified period, as is done in some of the eastern hospitals.

It is impossible to incorporate more than the bare framework of this extremely important subject within the narrow confines of a paper, but I wish to recapitulate some of the more important considerations in this paper, viz.: 1. Let your technique be extremely simple, but at the same time thorough. 2. Supervise the preparation as far as possible yourself, or entrust it to some one in whom you have absolute confidence. 3. Handle suppurating wounds or infectious cases just as little as possible, and when handled carry out with extreme care the rules of sterilization. 4. Pay more attention to mechanical sterilization than ever before, and when using chemicals, especially upon the hands, be sure that they do not leave an unsurgical field. 5. Observe closely the hands to see if they are free from abrasions and are in suitable condition to be thoroughly cleansed. 6. Scrupulously avoid if possible any carelessness in the technique, in the preparation and throughout the operation. 7. Take sufficient time to thoroughly complete the operation, thereby avoiding in many cases the necessity for drainage, but do not close any case in which you have a serious doubt as to advisability of drainage without draining.

In carrying out these precepts we come near to the attainment of perfection in technique, but all conscientious surgeons will tell you that in cases in which as far as they know all these rules were rigidly enforced, infection has followed with its serious results. The surgeon who has no suppuration and no fatalities is one not doing many operations, or else he is the prince of knaves and a worthy successor to Ananias of biblical record.

There are some factors, with whose nature we are yet unacquainted, capable of contributing to infection, and until we become familiar with them, we must not expect perfection, even though we seem in many instances to approach so close to this desired goal.

### BIBLIOGRAPHY.

The Aseptic Treatment of Wounds.—Schimmelbusch.

Dennis's System of Surgery.

The Evolution and Perfection of Aseptic Surgical Technique.—L. S. McMurtry.
